# Biocontrol of Non-*Saccharomyces* Yeasts in Vineyard against the Gray Mold Disease Agent *Botrytis cinerea*

**DOI:** 10.3390/microorganisms10020200

**Published:** 2022-01-18

**Authors:** Alice Agarbati, Laura Canonico, Tania Pecci, Gianfranco Romanazzi, Maurizio Ciani, Francesca Comitini

**Affiliations:** 1 Department of Life and Environmental Sciences, Marche Polytechnic University, Via Brecce Bianche, 60131 Ancona, Italy; a.agarbati@univpm.it (A.A.); l.canonico@univpm.it (L.C.); peccitania@gmail.com (T.P.); m.ciani@univpm.it (M.C.); 2 Department of Agricultural, Food and Environmental Sciences, Marche Polytechnic University, Via Brecce Bianche, 60131 Ancona, Italy; g.romanazzi@univpm.it

**Keywords:** bioactive yeasts, biocontrol, gray mold, *M. pulcherrima*, *A. pullulans*

## Abstract

Background: *Botrytis cinerea* (*B. cinerea*) is responsible for grape infection and damage to the winemaking and table grape sectors. Although anti-*Botrytis* chemicals are available, they are considered unsustainable for resistance phenomenon and adverse effects on the environment and human health. Research is focused on developing alternative approaches, such as exploiting biological control agents (BCAs). In this context, 19 yeasts of the genera *Cryptococcus*, *Aureobasidium*, *Metschnikowia*, *Kluyveromyces* and *Wickerhamomyces* were tested as antimicrobial agents against *B. cinerea* development. Methods: A combination of in vitro tests based on dual-culture methods, volatile organic compound production assay, laboratory tests on grape berries (punctured and sprayed with yeasts) and field experiments based on yeast treatments on grapes in vineyards allowed the selection of two potential BCAs. Results: *M. pulcherrima* DiSVA 269 and *A. pullulans* DiSVA 211 exhibited the best ability to contain the development of *B. cinerea*, showing the severity, the decay and the McKinney index lower than a commercial biological formulation consisting of a mixture of two different *A. pullulans* strains, which were used as positive controls. Conclusions: The results indicated that the selected strains were effective BCA candidates to counteract *B. cinerea* in the field, applying them in the partial or total replacement of conventional treatments.

## 1. Introduction

The fungus *Botrytis cinerea* (*B. cinerea*), also known as gray mold, is a natural component of grape microbiota [1] and is one of the main spoilage microorganisms that can cause consistent damage to crops worldwide [2,3]. *B. cinerea* occupies the second place in the world ranking of the top 10 pathogenic fungi in terms of diffusion and relative commercial loss, preceded only by *Magnaporthe oryzae* [4]. In the grape and wine industry, the impact of bunch rot is well established, because all cultivars are susceptible to this infection. A common way to counteract the development of *B. cinerea* is the application of chemical fungicides. Recently, it was introduced that a class of synthetic fungicides belongs to the succinate dehydrogenase inhibitors (SDHIs). Other chemicals, such as salts solutions recognized as safe (iron sulphate, ammonium bicarbonate, sodium silicate, sodium bicarbonate and sodium carbonate), are widely used to sanitise grapes surface. Ethanol vapours and other gas such as chlorine dioxide and ozone fumigation are also used, even if the sulphur dioxide remains the main method that is used [5].

These conventional anti-*Botrytis* treatments are considered unsustainable. Indeed, the frequent appearance of resistant strains, adverse effects of fungicides on the environment and human health and stuck fermentation using infected grapes have necessitated a new control strategy [3,5]. In this context, the development of complementary methods to synthetic agents, such as biological control agents (BCAs), could be considered an alternative approach to reducing gray mold [6,7,8,9]. However, only a few commercial products, based on fungal or bacterial genera, are available in Europe for the biological control of gray mold in vineyards [10]. Indeed, the applicability in cyclical seasons, different climatic trends and/or local agronomic conditions represent the actual limits to the full efficacy of natural treatments. Therefore, several studies have proposed natural microorganisms as effective, low-cost biological antagonists to counteract *B. cinerea* infection [6,8,9,10].

The use of yeast as a BCA offers some advantages, including the easy colonisation of dry surfaces for extended periods, simple nutrition requirements, rapid growth and potential antagonistic effects against pathogens [11]. Among the different antagonistic yeasts, *Metschnikowiapulcherrima* is a relevant yeast species that has been successfully applied to control pathogens of fruits and vegetables. The competition of *M. pulcherrima* for nutrients is the dominant mechanism during the biocontrol of *B. cinerea* [12]. In addition, *Wickerhamomycesanomalus* shows an antimicrobial activity against *B. cinerea* via multiple mechanisms, including the colonisation of wounds, a biological protective layer and the production of hydrolytic enzymes and volatile organic compounds [6]. Among other yeast genera, *Debaryomyces hansenii* [13], *Aureobasidium pullulans* [14], *Cryptococcus laurentii* [15] and *Sporidiobolus pararoseus* [16] have demonstrated the potential for the control of post-harvest decay of fruits and vegetables. In some cases, the attachment efficacy of yeasts depends on their abilities to secrete lytic enzymes, which might be involved in the biocontrol efficacy of antagonistic yeasts [17,18]. These antimicrobial properties of bioactive yeasts specifically change across genera and species and are affected by the yeast growth stage and the optimal yeast concentration [19].

The present study aimed to assess the effectiveness of 19 yeast strains belonging to five genera, isolated from natural environments, on *B. cinerea*. Preliminary screening was performed using an in vitro approach to select strains with a promising antimicrobial activity. The selected yeasts were evaluated in a vineyard to control the post-harvest decay of grapes before the grape harvest.

## 2. Materials and Methods

### 2.1. Yeasts and Culture Conditions

The yeasts used in the trials as BCAs against *B. cinerea* belonged to the Microbial Collection of the Department of Life and Environmental Sciences (DiSVA) of Polytechnic University of Marche (Italy). All yeasts reported in Table 1 were previously isolated from flowers, leaves and fruits.

All yeast strains were stored on yeast extract peptone dextrose (YPD) agar (yeast extract, 1%; peptone, 2%; dextrose, 2%; agar, 1.8%) at 4 °C before use. The commercial mold *B. cinerea* N51 (DSMZ Germany Collection of Microorganisms and Cell Cultures GmbH, Braunschweig, Germany) was maintained and stored at 4 °C on potato dextrose agar (PDA; Oxoid, Basingstoke, UK) until use. A commercial formulation named Botector^®^ (Manica S.p.a., Rovereto, Italy) was used as a bioactive positive control after reconstitution following the supplier’s suggestions (in 0.1% sterile water). This preparation was made up of freeze-dried cultures of two strains of *Aureobasidium pullulans*, as reported in the technical sheet according to Regulation (EC) No. 1907/2006.

### 2.2. Screening to Evaluate the Antagonistic Effects of Yeasts

Three different plate tests were set up to preliminarily determine the antimicrobial efficacy of yeasts against *B. cinerea*. In the first two cases, the competition for space and/or nutrients was simulated, while a contactless test was set up in the third experiment.

#### 2.2.1. Control of *B. cinerea* in an Early Stage of Infection

The ability of yeasts to control *B. cinerea* simulating an early stage of infection was assessed through an in vitro assay based on a dual-culture method, following the procedure described by Wang et al. [3] with some modifications. Firstly, yeast strains were precultured in an YPD broth for 48 h at 25 °C, and molds were grown on PDA for 96 h at 25 °C under sunlight exposure. Yeast cells were washed twice and resuspended in sterile water to a final concentration of 10^7^ cells/mL, which was obtained using a Thoma–Zeiss counting chamber [20]. *B. cinerea* spores were collected from a fresh plate with a sterile tip and diluted in a sterile 0.1% Triton X-100 solution to a final concentration of 10^5^ spores/mL. Ten microlitres of each suspension (yeast and mold) were simultaneously spotted on the PDA surface 5 cm apart. The test was performed in triplicate, and negative control was performed by inoculating 10 µL of sterile water, instead of the yeast suspension. The positive control was a 0.1% Botector^®^ (Manica S.p.a., Rovereto, Italy) solution adjusted to the final concentration similar to the other yeasts (10^7^ cells/mL). The plates were incubated at 25 °C for 5–7 days. The inhibitory effect of each yeast was evaluated by measuring the radial growth of *B. cinerea* in millimetres compared to the growth of mold in the absence of yeast and with that of the positive control.

#### 2.2.2. Containment of Advanced *Botrytis* Infection (Late)

The antagonistic activity of the same yeasts was evaluated through another in vitro assay based on a dual culture method, in which the mold was inoculated at the hyphal stage (simulation of advanced infection status). The test was carried out by streaking each yeast in a circular motion for 2 cm, starting from the edge toward the centre of the PDA plate medium. Together with a pre-incubation period of 24 h, this modality gave a double advantage to each yeast: it grew and released extracellular metabolites concentrated in the centre of the plate in the agar. After 24 h at 25 °C, 1 cm^2^ of *B. cinerea* mycelial plug (advanced development status) was placed in the centre of a Petri dish, 3 cm away from the yeast inoculum, and incubated for 5–7 days at 25 °C. Positive control was used, as described above. Three replicates were performed for each yeast/gray mold combination. The results were analysed by measuring the diameter (mm) of *B. cinerea* mycelial growth compared to the negative and positive controls.

#### 2.2.3. Antifungal Effect of Volatile Organic Compounds (VOCs) of Yeasts

The possible antimold effect of VOCs produced by yeast was evaluated following the method described by Oro et al. [12]. Briefly, each yeast was seeded on YPD agar medium and incubated for 24 h at 25 °C, and then, the lid of the Petri dish was substituted with a plate upside down (face to face without any direct contact between microorganisms) containing a PDA medium where a plug of *B. cinerea* actively grew. The two plates were sealed with a Parafilm and incubated at 25 °C for seven days. Growth inhibition was determined by measuring the diameter of the fungal mycelial growth (mm) compared to that of the negative control carried out without yeast culture. Positive control was performed using the commercial preparation Botector^®^ (Manica S.p.a., Rovereto, Italy), instead of the yeast culture. The test was performed in triplicate.

### 2.3. Extracellular Enzymatic Activities of Yeasts

All yeasts were characterised for their abilities to produce/secrete lytic enzymes with protease, esterase, amylase, β-glucosidase, cellobiose and killer activities. Protease, esterase and amylase activities were evaluated following the protocol described by Buzzini and Martini [21]. Briefly, the yeasts were spotted on a YEPG medium containing 2% casein, and a clear zone around the colony indicated the presence of the protease activity. The medium containing 1% peptone, 0.5% NaCl, 0.01% CaCl_2_·2H_2_O, 1% Tween-80 and 2% agar was used to assess the esterase activity through the presence of a visible precipitate around the yeast colony after incubation. Amylase activity was evaluated as starch hydrolysis by incubating the yeast on a medium containing soluble starch. After the yeast growth, the medium was flooded with an iodine solution, and a yellow zone around the colony indicated a starch-degrading ability. The evaluations of killer, cellobiose and β-glucosidase activities was carried out following the methods of Comitini et al. [22], Guo et al. [23] and Rosi et al. [24], respectively.

### 2.4. Antimicrobial Activity of Yeasts on Grape Berries

Based on the results obtained by plate assays, the six strains that showed the best antagonistic effects against *B. cinerea* were selected. These strains were then evaluated in harvested grape berries using two different methods. In both modalities, assays were carried out in batches (20 grapes per batch) previously sterilised in 1% sodium hypochlorite following the procedure proposed by Wang et al. [3]. In the first case, the berries of each batch were pierced with a sterile tip, causing a lesion that was lodged for the inoculation of the yeast suspensions (10 µL of 10^7^ cells/mL), followed by 10 µL of a suspension of 2 × 10^5^ spores/mL of *B. cinerea*. In the second modality, the berries were immersed in each yeast suspension; after 20 min of air drying, the *B. cinerea* suspension (2 × 10^5^ spores/mL) was sprayed over the berries. These were stored in a sterile plastic bag to simulate a humid environment, incubated for 20 days at 4 °C, then transferred to 25 °C for three days and observed for *B. cinerea* growth.

After the two experimental preparations, the berries were monitored daily for three days to observe the damage caused by *B. cinerea* infection. Disease severity was recorded according to an empirical scale with six degrees: 0, healthy fruit; 1, 1–20% fruit surface infected; 2, 21–40% fruit surface infected; 3, 41–60% fruit surface infected; 4, 61–80% fruit surface infected; and 5, ≥81% fruit surface infected. The infection index or McKinney index incorporates both the incidence and severity of the disease and expresses the weighted means of the disease as a percentage of the maximum possible level [25]. Specifically, it was calculated using the following equation:

I = d × f/N × D × 100,

where d is the category of the rot intensity scored on the grapes, f is its frequency, N is the total number of grapes examined (healthy and rotted), and D is the highest category of the disease intensity that occurs on an empirical scale [12].

### 2.5. Evaluation of Two Selected Bioactive Yeasts by Field Experiments

Based on the results of experiments on grapes, two antagonistic yeast strains were selected. The efficacy of the two antagonists against bunch rots of wine grapes was evaluated during the 2018 harvest in a vineyard located in the centre of Italy. Untreated and Botector^®^-treated vines served as negative and positive controls, respectively. The two antagonistic strains were tested separately and together in a mixture (MIX) at a 1:1 volume ratio. Initially, the biomass of the two yeasts was produced in a 30 L bioreactor and harvested after ultracentrifugation at 4 °C under sterile conditions. The biomass was dissolved in sterile water and aliquoted (at a final concentration of 1 × 10^7^ cells/mL) into three lots of 5 L with strains 1, 2 and MIX, respectively.

The experimental plots consisted of one to nine vines per treatment in the different experiments, arranged as randomised blocks with five replicates. The two bioactive strains, the MIX and the controls were applied until run-off, with a motor-driven back-sprayer, in two stages: (i) at the time of the bunch veraison; and (ii) at the time of grape maturity. After each application, representative grape samples were collected to test yeast colonisation in the laboratory through viable cell counts using WL nutrient agar (Wallerstein Laboratories, Oxoid, Hampshire, UK) with 0.02% biphenyl to prevent indigenous mold diffusion. Based on its differential properties, this media allowed us to distinguish *M. pulcherrima* and *A. pullulans* species from other indigenous yeast species even if it was not possible to distinguish native strains from those sprayed belonging to the same specie *M. pulcherrima* colonies appearing as red colonies, whereas *A. pullulans* appeared as a “branched star” colony. At the harvest time, according to the farmer, all treated bunches were not collected to evaluate the antagonistic effect of yeasts sprayed by monitoring viable plate counts until the 10th day. Natural infection with *B. cinerea* was expected.

### 2.6. Statistical Analyses

The data were analysed using one-way analysis of variance (ANOVA), followed by Duncan’s test (*p* < 0.05). The percentage data were arcsine-transformed before analysis to improve the homogeneity of variance, when the range of percentages was >40%. The actual values were also presented. All trials were repeated at least twice with at least three replicates. Data from two or more experiments were pooled, and statistical analysis to determine the homogeneity of variances was performed using Levene’s test.

## 3. Results

### 3.1. In Vitro Antagonistic Effect of Potential BCAs

**Figure 1:**
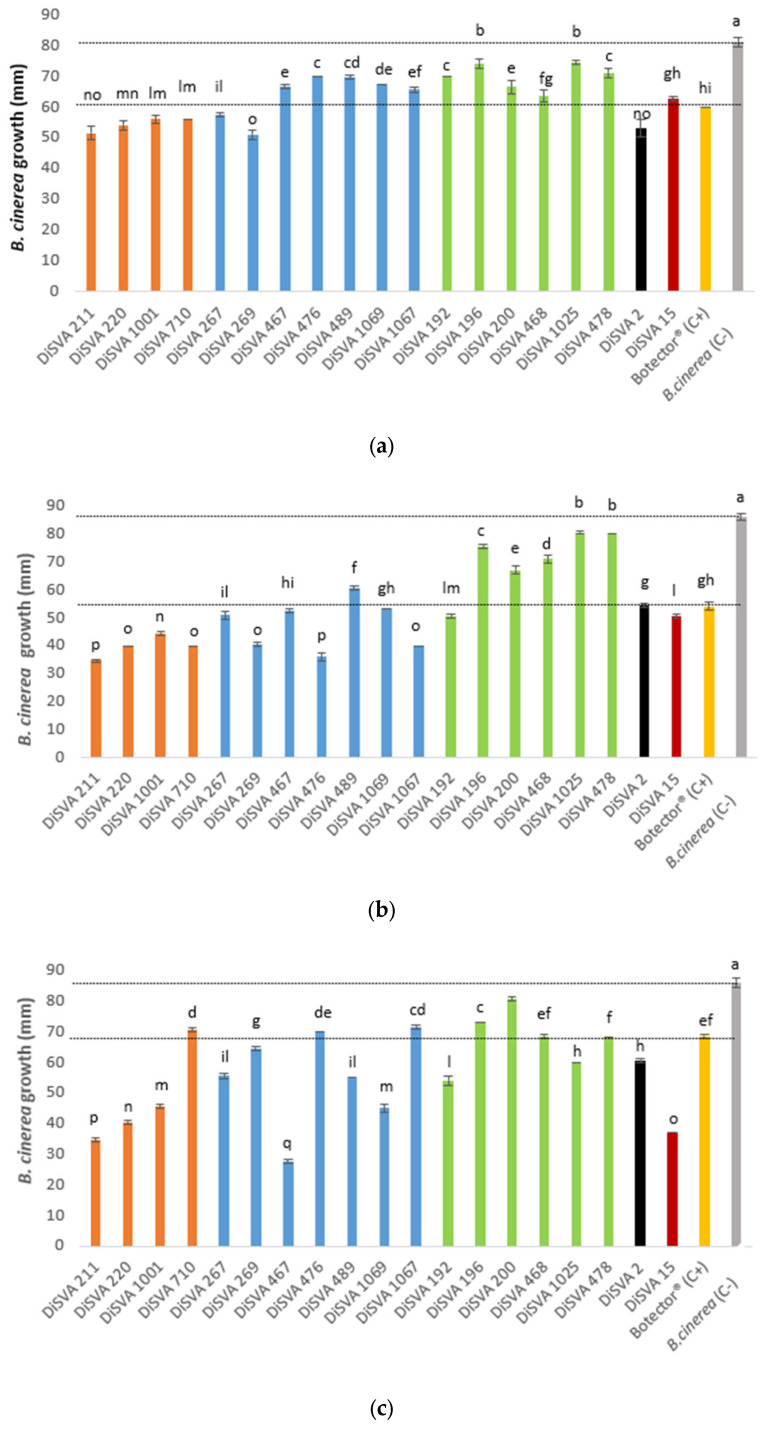
Antagonistic activity of yeasts against *Botrytis cinerea* (*B. cinerea*) growth compared with the negative and positive controls: (**a**) trials carried out considering an early stage of mold infection; (**b**) trials carried out considering an advanced stage of mold infection. (**c**) yeast’s volatile organic compounds (VOCs) antimicrobial activity on *B. cinerea* infection. The dotted lines highlighted the positive and negative control values, and the different superscript letters (^a, b, c, d, e, f, g, h, I, l, m, n, o, p^) among the bars are significantly different, according to Duncan’s test (*p* < 0.05).

The abilities of the 19 potential BCAs were preliminarily tested in three plate assays. First, the possibility of yeasts carrying out a control action at the early stage of *B. cinerea* infection was tested using two co-inoculated suspensions of yeast and mold spores. The results of this assay are shown in Figure 1a.

All strains tested showed a significant ability to control the development of mold than the negative control, in which a drop of sterile water was placed in the plate. However, different degrees of containment were observed. In particular, all *A. pullulans*, *M. pulcherrima* DiSVA 267 and DiSVA 269 strains, *W. anomalus* DiSVA 2 and *Kluyveromyces wickerhamii* DiSVA 15 showed a reduction in the mold growth of approximately 30 mm when compared with the negative control (hyphae radial growth of negative control = 80 mm), and an additional 10 mm of reduction compared with the positive control Botector^®^ (hyphae radial growth = 60 mm).

Similarly, all potential BCAs showed the ability to inhibit the growth of *B. cinerea* in an advanced stage of infection (simulated with an inoculum of a plug with germinated mold) compared to the negative control, which showed a hyphal growth of approximately 85 mm (Figure 1b). In this thesis, the positive control reduced the mold hyphae growth by approximately 30 mm and all *A. pullulans*, all *M. pulcherrima* (apart from DiSVA 489 strain), *W. anomalus* DiSVA 2, *K. wickerhamii* DiSVA 15 and only *C. albidus* DiSVA 192 showed a better or equal reduction in *B. cinerea* hyphal growth. In particular, *A. pullulans* DiSVA 211, *M. pulcherrima* DiSVA 269 and DiSVA 1067 reduced the mold growth by approximately 50 mm.

Finally, Figure 1c shows the results of the test that assayed the possible mechanism of the control mediated by VOC production. In this test, the positive control reduced the mold growth by 15 mm compared with the negative control, while almost all potential BCA strains tested showed better results than the positive control (Botector^®^). *A. pullulans* DiSVA 211 and DiSVA 220, *M. pulcherrima* DiSVA 467 and *K. wickerhamii* DiSVA 15 allowed the development of approximately 40 mm of *B. cinerea* with a reduction of approximately 50 mm in comparison with the negative control and an additional growth reduction of approximately 30 mm in comparison with the positive control Botector^®^.

### 3.2. Extracellular Enzymatic Activities of Potential BCAs

The 19 yeast and yeast-like strains were also evaluated for extracellular lytic enzyme production and killer activity, and the results are reported in Table 2.

All *A. pullulans* strains exhibited protease, β-glucosidase, amylase, cellobiose and esterase activities, except DiSVA 220 and DiSVA 710 strains that did not show a protease activity. In contrast, none of the *A. pullulans* strains exhibited a killer ability against the sensitive yeast. A greater variability was observed among the *M. pulcherrima* strains. All strains produced cellobiosidase enzymes but did not show killer and amylase activities. Only *M. pulcherrima* DiSVA 269 also exhibited an esterase activity, while DiSVA 269 and DiSVA 476 showed a β-glucosidase activity, and DiSVA 467 produced protease lytic enzymes. All the *Cryptococcus* strains exhibited amylase, cellobiose (except DiSVA 196) and esterase activities. Protease was detected only by DiSVA 192, DiSVA 196 and DiSVA 468, whereas no β-glucosidase and killer activities were observed. *K. wickerhamii* DiSVA 15 showed only cellobiose and killer activities, whereas *W. anomalus* DiSVA 2 also produced esterase enzymes.

### 3.3. Evaluation of the BCAs Effectiveness on Grapes

From a comparative analysis of the results obtained in the three plate tests (Figure 1a–c), the strains showing a significant control action superior to that of Botector^®^ were selected and subsequently tested in the harvested berries in the laboratory. Seven strains of *A. pullulans*, DiSVA 211, DiSVA 220, DiSVA 1001, *M. pulcherrima* strains DiSVA 267 and DiSVA 269 and *W. anomalus* DiSVA 2 were evaluated.

**Figure 2:**
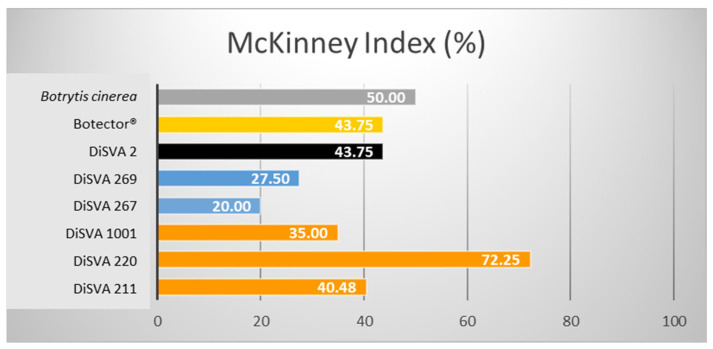
McKinney indices (%) of the six potential bioactive yeasts on lesioned grape berries.

To be used as a BCA in post-harvest applications, microorganisms should not have phytotoxic effects besides being effective against post-harvest pathogens. Artificially wounded berries treated with yeast did not show any evidence of phytotoxic effects for 25 days, because the wound size was not different from that of the negative control (data not shown). In addition, fruit sprayed with a yeast solution or inoculated in a berry wound, cold-stored and then exposed to shelf life did not show any phytotoxic effect. Thus, the pre-requisite for the possible consideration of these three strains of biocontrol agents was fulfilled. After the post-harvest treatment of lesioned berries, the damage caused by *B. cinerea* on the berries and the relative effectiveness of the seven selected BACs were evaluated based on the percentage of decay (Table 3) and the McKinney index (Figure 2).

All strains effectively reduced the gray rot in grape berries during storage after post-harvest treatments. Lesioned fruits showed a significantly reduced decay compared with untreated controls, with higher effectiveness than Botector^®^. Indeed, after a rank analysis, all the strains were placed in a more favourable position than the Botector^®^ (rank 6), except for *W. anomalus* DiSVA 2, which seemed to be the least effective (rank 7). *A. pullulans* DiSVA 211 proved to be the most effective in controlling the damage spread, placing it in the first rank.

A reduction in the McKinney index was evident in all strains compared to in the control. These antimicrobial action results were well correlated with each other: the two strains of *M. pulcherrima* and *A. pullulans* DiSVA 211 showed a more significant damage reduction and, therefore, a greater efficacy. Again, the *W. anomalus* strain DiSVA 2 showed the lowest control efficacy.

**Figure 3:**
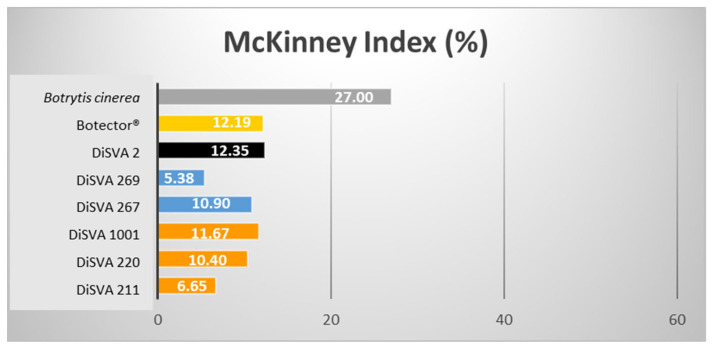
McKinney indices (%) of the six potential bioactive yeasts on surface-sprayed harvested grape berries.

Similar results were obtained from the same experiment on berries not lesioned but sprayed with a suspension of the same BCAs (Table 4 and Figure 3).

In this case, the best decay control was achieved by *M. pulcherrima* strain DiSVA 269 (rank 1 in the decay control). This strain confirmed its best behaviour showing a McKinney index of 5.38%, followed by *A. pullulans* strain DiSVA 211 with a McKinney index of 6.65%. As expected, the colonisation level of *B. cinerea* in damaged grapes was higher (McKinney index of 50%) than that in sprayed grapes (McKinney index of 27%). Consequently, the abilities of these selected BCAs to control gray mold were also lower in unpunctured grapes but not less effective.

### 3.4. Application of M. pulcherrima DiSVA 269 and A. pullulans DiSVA 211 in the Field

After a comprehensive analysis of all the results obtained, the strains which were most effective in controlling the development of *B. cinerea* in laboratory tests (both in plates and on grapes) were *M. pulcherrima* DiSVA 269 and *A. pullulans* DiSVA 211. Therefore, these two strains were used in the field during the pre-harvest treatment. In the first evaluation, after each field treatment (both at the veraison time and at the ripening of the bunches), the real colonisation of the two strains, used both separately and together in the MIX, was evaluated. The results reported in Table 5 showed the actual and stable colonisation of both yeasts for each treatment.

Ten days after treatment, the persistence results confirmed that both yeasts maintained the same viability level (Table 5).

**Figure 4:**
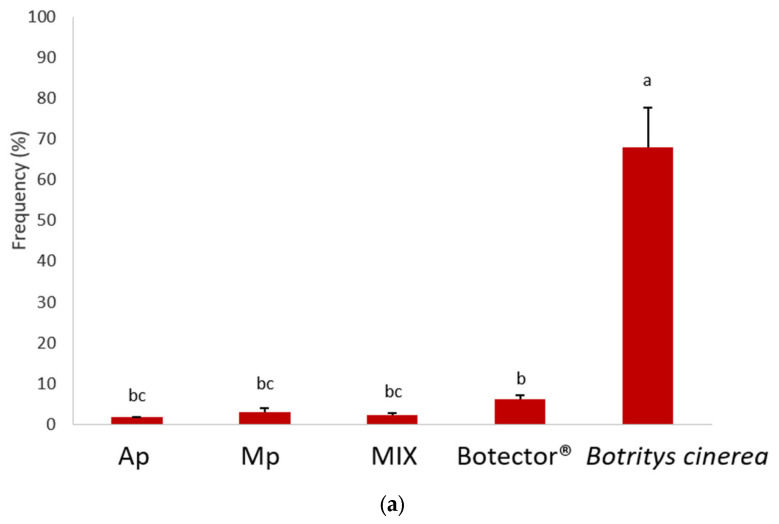

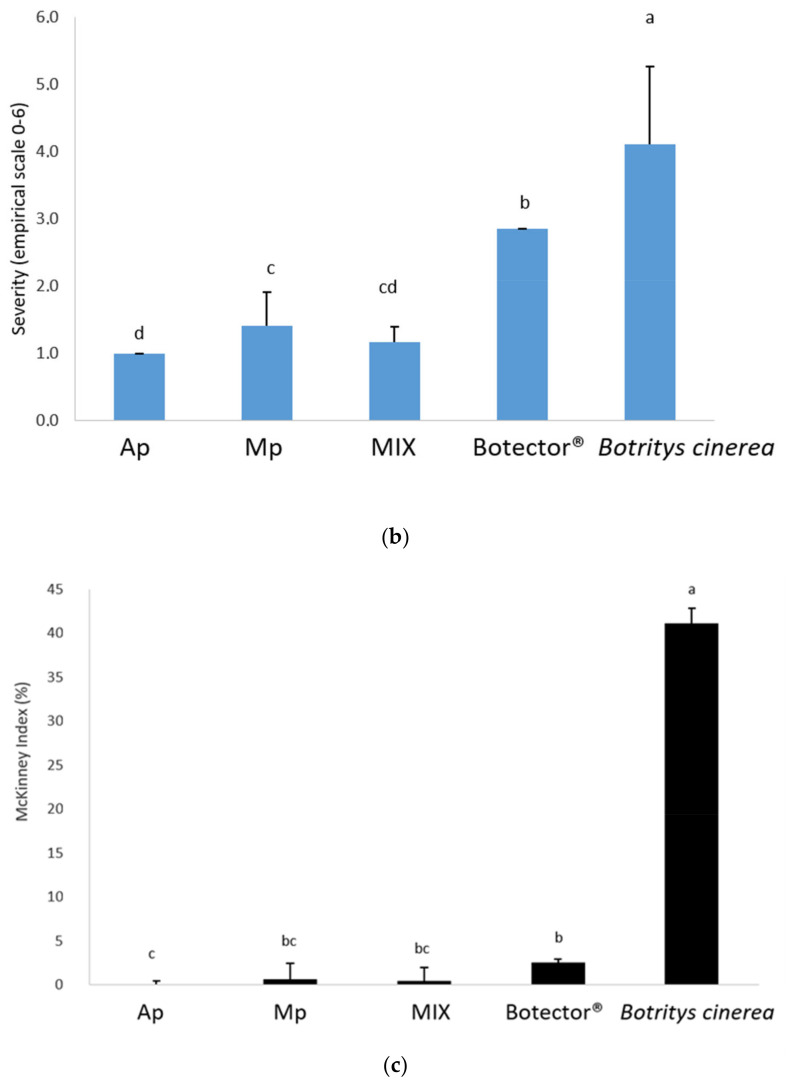
Frequency (**a**), decay (**b**) and McKinney’s index (**c**) of gray mold on grapes. Different superscript letters (^a, b, c, d^) show significant differences according to Duncan’s test (*p* < 0.05).

The effectiveness of post-harvest treatments (shelf life at room temperature) was evaluated by harvesting the berries 24 h after the second treatment and 10 days later. The growth of *B. cinerea* was evaluated by the statistical analysis of the randomised block frequency, the severity and the McKinney index (Figure 4). The results showed that *B. cinerea* grew on most of the berries monitored in the untreated samples.

The thesis treated with Botector^®^ showed good mold control, although with nonhomogeneous data in the various samples. In the thesis Mp, *M. pulcherrima* DiSVA 269 showed a very low incidence of damage, but in berries presumably not colonised by BCA, *B. cinerea* grew considerably. *A. pullulans* strain DiSVA 211 showed a lower control of mold decay, but with more homogeneous results for all samples.

Finally, the MIX thesis exhibited a higher control capacity, combining the positive aspects of the two yeasts obtained separately. Indeed, the severity and the McKinney index were significantly lower in the latter case.

## 4. Discussion

Currently, there is increasing interest in using BCAs to suppress bunch rot caused by *B. cinerea* in grapes. Different filamentous fungi, bacteria and yeasts have been selected as potential biological suppressors of this pathogen [26,27]. Many of these suppressors suppress the growth of plant pathogens through competition for nutrients or by the production of inhibitory metabolites and/or parasitism. Although several small-scale studies have been carried out highlighting different antagonist strains, and for some of them, the mechanisms of action have been understood and the practical application of BCAs remains an unsolved problem. This difficulty is probably linked to the commercialisation of BCAs, which require a multistep process that involves the isolation and screening of potential antagonism, testing the efficiency of the isolate in the field, biomass production, formulation, toxicity studies, delivery, compatibility, registration and release [28]. However, the evidence that in vivo studies on antagonistic yeasts play efficient control actions in a large variety of vegetal matrices, including grapes, prompted us to research this area.

In this study, 19 strains, previously characterised as antagonists, were evaluated for their potential bio-preservative effects against *B. cinerea* in wine grapes. 

Preliminary findings, carried out in plate assays, indicated that *M. pulcherrima*, *W. anomalus* and *A. pullulans* are yeast species involved in gray rot disease control, supporting previous results [3,4,6,29,30]. The antimicrobial activity of the two yeasts revealed under laboratory conditions, together with the results of the colonisation and persistence in a vineyard, indicates that the control of disease (diffusion and severity) is due to sprayed yeasts on grapes.

In this regard, several mechanisms of action are suggested. *M. pulcherrima* strain MPR3, isolated from spontaneous olive fermentation, has been demonstrated to have a great antifungal activity against *B. cinerea*, mediated by VOC production [31], while other strains appear to effectively control gray rot with other mechanisms associated with iron depletion [29,32]. Although the mechanism by which yeasts inhibit the development of *B. cinerea* has not been studied here, our preliminary results suggested that *M. pulcherrima* DiSVA 269 exerts its inhibitory action in three possible ways. The first could be the competition for nutrients. Indeed, it colonises grape berries and persists on grape berries for at least 10 days after treatment.

The second strategy could be the presence of β-glucosidase activity, which has been well studied as a potential cause of fungal wall damage [33]. In particular, after enzymatic lytic evaluation, *M. pulcherrima* strain DiSVA 269 was selected to be more effective in controlling *B. cinerea*. The third way *M. pulcherrima* strain DiSVA 269 controls gray rot could be due to pulcherriminic acid production that depletes iron present in the environment, making it unavailable to other microorganisms, as previously reported in an in vitro study [34].

Multiple modes of action of *A. pullulans* have been reported to explain its biocontrol efficacy, including competition for nutrients and space, production of cell wall-degrading enzymes, synthesis of antifungal compounds and mycoparasitism [2]. In this study, the evaluation of the principal enzymatic activities showed that all strains of *A. pullulans* tested were positive for all plate tests. This trend confirmed the ability of this species to produce and secrete different lytic enzymes [35].

*A. pullulans* is also known for inhibiting mold development by competing for space [2,35,36]. The results showed the ability to persist on the surface of the grapes for at least 10 days after treatment, and in the case of damaged grapes, *A. pullulans* DiSVA 211 showed the lowest decay percentage. The mechanism of action could be due to the expansion of this yeast-like structure in the wound, limiting the colonisation and development of *B. cinerea*. This inhibitory action can also be reinforced through VOC production, as reported by Don et al. [37]. On the other hand, *A. pullulans* is known to exhibit a high genotypic diversity [2]; therefore, the choice of strains to be used as biocontrol agents is a critical step. Indeed, among the four *A. pullulans* strains tested here, only two were more effective in plate tests, and only DiSVA 211 confirmed their significant efficacies in vivo during field treatments. Despite the availability of *A. pullulans*-based products currently marketed, such as Botector^®^ or BIO-FERM [38,39,40], new candidates to control gray rot in vineyards are required. In this regard, the results of field treatments indicated that several factors should be evaluated, such as the fast colonisation of potential BCAs and their persistence, which are characteristic of *M. pulcherrima* DiSVA 269 and *A. pullulans* DiSVA 211, selected as the best potential BCAs. In this regard, the MIX trial showed promising results in gray mold inhibition, expressed as the frequency, the severity and the McKinney index, probably due to the synergistic action of competition for space and nutrients, specific enzymatic activities and production of VOCs exerted by both species.

Further investigations of the anti-*B. cinerea* by two selected yeasts, in pure and mixed applications and under different agronomical and environmental conditions, are needed to constitute a promising source of knowledge and to set up strategies to prevent or reduce harvested commodity damage.

## 5. Conclusions

In this study, *A. pullulans* and *M. pulcherrima* were the most promising potential BCAs for the development of *B. cinerea* mold. The trials carried out in vineyards showed their anti-*B. cinerea* action and could be proposed as a single species or in combination by exploiting the synergistic action of their antagonistic capacities through the rapid colonisation of the grapes and persistence on the grape surface. This approach meets consumer expectations and widely accepts the development of bio-based applications to exert microbial control in agro-food chains, according to eco-friendly approaches and products free of synthetic chemicals. For these purposes, the approach proposed here could be further investigated to understand the mechanisms of action and evaluate the safety of the proposed bioactive yeasts.

## Figures and Tables

**Table 1:** Species and origins of the yeast strains tested as potential antifungal agents.

Species	Strain	Origin
*Aureobasidium pullulans*	DiSVA 211	Flowers
	DiSVA 220	Flowers
	DiSVA 1001	Montepulciano grape
	DiSVA 710	Montepulciano grape
*Metschnikowia pulcherrima*	DiSVA 267	Grape
	DiSVA 269	Grape
	DiSVA 467	Verdicchio grape
	DiSVA 476	Verdicchio grape
	DiSVA 489	Verdicchio grape
	DiSVA 1069	Verdicchio grape
	DiSVA 1067	Montepulciano grape
*Cryptococcus albidus*	DiSVA 192	Flowers
	DiSVA 196	Red berries
	DiSVA 200	Leaves
*Cryptococcus magnus*	DiSVA 468	Verdicchio grape
*Cryptococcus carnescens*	DiSVA 1025	Montepulciano grape
*Cryptococcus* sp.	DiSVA 478	Verdicchio grape
*Wickerhamomyces anomalus*	DiSVA 2	Sourdough
*Kluyveromyces wickerhamii*	DiSVA 15	Ripened fruit

**Table 2:** Enzymatic characterisation of potential bioactive yeasts.

Species	Strain	Production of Lytic Enzymes					
		Protease	β-Glucosidase	Amylase	Cellobiosidase	Esterase	Killer
*Aureobasidium pullulans*	DiSVA 211	+	+	+	+	+	-
	DiSVA 220	-	+	+	+	+	-
	DiSVA 1001	+	+	+	+	+	-
	DiSVA 710	-	+	+	+	+	-
*Metschnikowia pulcherrima*	DiSVA 267	-	-	-	+	-	-
	DiSVA 269	-	+	-	+	+	-
	DiSVA 467	+	-	-	+	-	-
	DiSVA 476	-	+	-	+	-	-
	DiSVA 489	-	-	-	+	-	-
	DiSVA 1069	-	-	-	+	-	-
	DiSVA 1067	-	-	-	+	-	-
*Cryptococcus albidus*	DiSVA 192	+	-	+	+	+	-
	DiSVA 196	+	-	+	-	+	-
	DiSVA 200	-	-	+	+	+	-
*Cryptococcus magnus*	DiSVA 468	+	-	+	+	+	-
*Cryptococcus carnescens*	DiSVA 1025	-	-	+	+	+	-
*Cryptococcus* sp.	DiSVA 478	+	+	+	+	+	-
*Wickerhamomyces anomalus*	DiSVA 2	-	-	-	+	+	+
*Kluyveromyces wickerhamii*	DiSVA 15	-	-	-	+	-	+

**Table 3:** Decay percentage (%) and relative rank analyses of the six potential bioactive yeasts on lesioned grape berries.

Species	Strain	Decay (%)	Rank
*Aureobasidium pullulans*	DiSVA 211	5.9	1
*Aureobasidium pullulans*	DiSVA 220	7.68	2
*Metschnikowia pulcherrima*	DiSVA 267	8.25	3
*Metschnikowia pulcherrima*	DiSVA 269	10.6	4
*Aureobasidium pullulans*	DiSVA 1001	14.8	5
*Aureobasidium pullulans* (C+)	Botector^®^	16.69	6
*Wickerhamomyces anomalus*	DiSVA 2	18.91	7
*Botrytis cinerea* (C−)	N51	23.06	8

**Table 4:** Decay (%) and relative rank analyses of the six potential bioactive yeasts on surface-infected grape berries. The *B*. *cinerea* growth was observed after three days at 25 °C.

Species	Strain	Decay (%)	Rank
*Metschnikowia pulcherrima*	DiSVA 269	12.00	1
*Metschnikowia pulcherrima*	DiSVA 267	12.87	2
*Aureobasidium pullulans*	DiSVA 1001	16.65	3
*Aureobasidium pullulans*	DiSVA 211	17.47	4
*Aureobasidium pullulans*	DiSVA 220	18.43	5
*Aureobasidium pullulans* (C+)	Botector^®^	20.15	6
*Wickerhamomyces anomalus*	DiSVA 2	26.05	7
*Botrytis cinerea* (C−)	N51	36.32	8

**Table 5:** Evaluation of the colonization of the potential biological control agents (BCAs) on the grapes after each treatment and their persistence. Values are reported as total microflora counts, while the values inside the brackets represented the *A. pullulans* DiSVA 211 (Ap)- and *M. pulcherrima* DiSVA 269 (Mp)-inoculated species without the distinction between indigenous and inoculated strains. Data are represented as means ± standard deviations.

	Yeast Colonization (log CFU/mL)				Yeast Persistence (log CFU/mL)
Trials	Bunches Veraison		Bunches Maturity		10th Day after Ripening
	Bf *	Af **	Bf	Af	
Ap	2.4.30 ± 0.01	4.68 ± 0.09 (Ap: 4.27 ± 0.12)	4.96 ± 0.21 (Ap: 3.69 ± 0.21)	4.99 ± 0.13 (Ap: 4.17 ± 0.08)	5.43 ± 0.11 (Ap: 4.93 ± 0.08)
Mp	4.63 ± 0.03	4.64 ± 0.20 (Mp: 4.23 ± 0.15)	4.61 ± 0.11 (Mp: 2.87 ± 0.02)	4.73 ± 0.17 (Mp: 4.14 ± 0.30)	5.38 ± 0.19 (Mp: 4.83 ± 0.05)
MIX	4.86 ± 0.15	4.94 ± 0.13 (Ap: 4.14 ± 0.07; Mp: 2.07 ± 0.01)	5.51 ± 0.09 (Ap: 0.00 ± 0.00; Mp: 4.20 ± 0.07)	5.20 ± 0.18 (Ap: 4.17 ± 0.08; Mp: 4.27 ± 0.29)	5.36 ± 0.21 (Ap: 4.97 ± 0.06; Mp: 4.55 ± 0.23)
Botector^®^	4.64± 0.02	4.75 ± 0.01 (Ap: 4.11 ± 0.23)	4.92 ± 0.21 (Ap: 0.00 ± 0.00)	4.79 ± 0.19 (Ap: 3.84 ± 0.25)	5.44 ± 0.04 (Ap: 5.04 ± 0.03)
Untreated	4.79 ± 0.07	4.57 ± 0.05	4.97 ± 0.07	4.77 ± 0.09	5.53 ± 0.31

* Bf, before treatment; ** Af, after treatment. Indeed, significant increases in the concentrations of *M. pulcherrima* and *A. pullulans*, compared with the epiphytic population (which showed average expected values), were observed after each treatment, reaching levels of approximately 4.30 log CFU/mL at the veraison of each yeast (Ap and Mp). In the case of the MIX thesis, the proportions of the two yeasts were maintained, indicating the excellent coexistence of the two strains within the mixture. The concentrations of the yeasts applied during the veraison application suffered only a slight decline, more in *M. pulcherrima,* which was reduced to 2.87 log CFU/mL until the second treatment (bunch maturity, before treatment). However, in the second treatment (bunches maturity, after treatment), the order of magnitude of each yeast increased by almost 1 logarithmic order, returning to log4 (higher in the MIX thesis). This preliminary result established the real and stable colonisation of each selected yeast on grapes under real conditions in the field.
